# A non-field analytical method for solving problems in aero-acoustics

**DOI:** 10.1038/s41598-020-76687-x

**Published:** 2020-11-12

**Authors:** Vladimir Kulish, Jiří Nožička, Jakub Suchý

**Affiliations:** Department of Fluid Mechanics and Thermodynamics, Faculty of Mechanical Engineering, Czech Technical University in Prague, Technická 4, 166 07 Prague 6, Czech Republic

**Keywords:** Engineering, Mechanical engineering

## Abstract

In 2000, a non-field analytical method for solving various problems of energy and information transport has been developed by Kulish and Lage. Based on the Laplace transform technique, this elegant method yields closed-form solutions written in the form of integral equations, which relate local values of an intensive properties such as, for instance, velocity, mass concentration, temperature with the corresponding derivative, that is, shear stress, mass flux, temperature gradient. Over the past 20 years, applied to solving numerous problems of energy and information transport, the method—now known as the method of Kulish—proved to be very efficient. In this paper—for the first time—the method is applied to problems in aeroacoustic. As a result, an integral relation between the local values of the acoustic pressure and the corresponding velocity perturbation has been derived. The said relation is valid for axisymmetric cases of planar, cylindrical and spherical geometries.

## Introduction

Nowadays the analytical theory of energy transport processes is developing rapidly. A result, presented by means of a simple formula, is always more preferable than a solution obtained numerically, especially in the case, when the found analytical solution is part of a “larger system”. Moreover, namely analytical methods are one of the main sources for the improvement and development of numerical methods, as well as for drawing general theoretical conclusions.

In 2000, a non-field analytical method for solving various problems of energy and information transport has been developed by Kulish and Lage^1^. Based on the Laplace transform technique, this elegant method yields closed-form solutions written in the form of integral equations, which relate local values of an intensive properties such as, for instance, velocity, mass concentration, temperature with the corresponding derivative, that is, shear stress, mass flux, temperature gradient.

It is worth noting that solutions, obtained by the said method, are valid everywhere within the domain of interest and, which is of extreme importance, remain valid on the domain boundaries.

Over the past 20 years, applied to solving numerous problems of energy and information transport, the method—now known as the method of Kulish—proved to be very efficient. In particular, the method was employed to solving various problems of transient diffusion^2^ and was then generalised by Frankel for tackling problems within finite domains^3^. The method has been extended even further to render analytical solutions to problems in micro- and nano-scale heat transfer based on the dual-phase-lag model proposed by Tzou^4^ as well as problems in ultra-fast heat transfer described by the hyperbolic heat conduction equation with no heat source/sink^5,6^ and moving boundaries^7^. Then solutions with the heat source/sink have been presented in^8,9^. Finally, the same very method has been presented in its most generalised form in^10^.

In this paper—for the first time—the method is applied to problems in aeroacoustic. As a result, an integral relation between the local values of the acoustic pressure and the corresponding velocity perturbation has been derived. The relation is valid for axisymmetric cases of planar, cylindrical, and spherical geometries.

The following section of the paper provides the problem formulation, which is in line with classical acoustic analogies^11–21^. The detailed solution procedure for the case of a zero sound source acoustic function is presented in “Solution procedure: the case of no source”. The sound source function in the form of the Fourier series is then incorporated in “Incorporation of the sound source function”. Such a choice of the source function is justified by the fact that the Fourier series is the most convenient way of presenting periodic functions, which most often arise as a result of vibrations. Note also that many other functions, including some functions, which are not continuous, can be expressed in the form of the Fourier series. The results of the model validation are shown in “Model validation”, in which the choice of examples has been dictated, first, by examples considered within known acoustic analogies,and, second, by the need to consider the most representative physical situations, such as, for instance, periodic boundary conditions or pulses (here modelled as the Gaussian functions).

## Problem formulation

In order to determine the sound pressure level, defined as

1 $$SPL=20{log}_{10}\frac{{\widehat{p}}_{RMS}}{{p}_{ref}}{\text{dB}}$$

where $\widehat{p}$ is the acoustic pressure and the reference sound pressure is usually ${p}_{ref}=20$ μPa, one needs to know the pressure field *p*.

In general, the pressure field is modelled by the wave equation

2 $$\frac{{\partial }^{2}p}{\partial {t}^{2}}-{c}^{2}{\nabla }^{2}p=S\left(\overline{x},t\right)$$

where *c* is the speed of sound and *S* denotes the sound source function^11–21^.

Depending on how the sound source function is modelled, different aero-acoustic models—known as acoustic analogies—exist. Among them are the Lighthill^11,12^, Powel^13^, Ffowcs Williams-Hawkings^14^ analogies, and some others^15–21^.

## Solution procedure: the case of no source

The elementary case of the homogeneous wave equation, considered in this section, will be used as a building block to obtain more complex solutions with the source function.

Three (and only three) geometries of the boundary allow a reduction from three to one in the number of spatial coordinates needed to describe energy transport through the medium. Three different values of a geometric factor γ are used to characterise these simplifying geometries, namely: the infinite plane, γ = 1/2; the infinitely long cylinder, γ = 0; and the sphere, γ = − 1/2 (see^9^ for details).

These geometries simplify the Laplacian operator so that Eq. (2), in which $S(\overline{x},t)\equiv 0,$ acquires a simpler form

3 $$\frac{1}{{c}^{2}}\frac{{\partial }^{2}p(r,t)}{\partial {t}^{2}}=\frac{{\partial }^{2}p(r,t)}{\partial {r}^{2}}+\frac{1-2\gamma }{r+R}\frac{\partial p(r,t)}{\partial r}$$

The medium is initially at equilibrium with the transient response that ensues on perturbing the system. It is convenient to define *t* = 0 as the last instant at which equilibrium exists. Thus, the initial condition is defined as

4 $${\left.p\left(r\right)\right|}_{t=0}={p}_{0}$$

where *r* is the spatial coordinate directed normal to the boundary and having its origin at the boundary surface. In the cases of cylindrical and spherical geometries, the *R* in Eq. (3) represents the radius of the surface,*R* is without significance in the planar case.

A final restriction is that only times *t* are considered, which are short enough that the perturbation, which started at *t* = 0 at the planar, cylindrical, or spherical boundary has not yet reached any other boundary of the medium. Alternatively, *t* can be considered to be unrestricted provided that all other boundaries are located infinitely far from the boundary of interest. In that event, the value of the acoustic pressure *p* at a point infinitely remote from *r* = 0 will remain unaltered after any finite time, so that

5 $${\left.p\left(r\right)\right|}_{x=\infty }={p}_{0}$$

Consideration will be restricted to perturbations, which are imposed on the medium through one of its boundaries. The condition on the boundary, *r* = 0, is deliberately not imposed. This will become clear from the following solution procedure. It suffices to mention here that the boundary condition may be of the Dirichlet, Neumann, Cauchy, or any other type^22^.

Upon introducing the excess acoustic pressure $\widehat{p}(r,t)=p(r,t)-{p}_{0},$ Eq. (3) becomes

6 $$\frac{1}{{c}^{2}}\frac{{\partial }^{2}\widehat{p}\left(r,t\right)}{\partial {t}^{2}}=\frac{{\partial }^{2}\widehat{p}\left(r,t\right)}{\partial {r}^{2}}+\frac{1-2\gamma }{r+R}\frac{\partial \widehat{p}\left(r,t\right)}{\partial r}$$

with the initial and boundary conditions

7 $${\left.\widehat{p}\left(r\right)\right|}_{t=0}={\left.\widehat{p}\left(r\right)\right|}_{t=\infty }=0.$$

It is worth noting that the method, presented here, is based on the Laplace transform technique (see the following solution procedure). When applied to partial differential equations, this technique leads to the appearance of extra terms in the transforms of the time derivatives, unless the relevant initial conditions are zero. In the present case, introducing the excess pressure $\widehat{p} \left(r\right)$ does not change the differential equations involved, but eliminates the necessity to carry on extra terms in the Laplace space. Once the solution has been obtained, the original variables are restored and the reference pressure ${p}_{0}$ is added to the final result. Note also that, as regards the physics of the problem in question, a constant reference pressure, added to or subtracted from the entire pressure field does not involve any change whatsoever.

Taking the Laplace transform of Eqs. (6) and (7) results into

8 $$\frac{{d}^{2}\Pi (r;s)}{d{r}^{2}}+\frac{1-2\gamma }{r+R}\frac{d\Pi (r;s)}{dr}={\left(\frac{s}{c}\right)}^{2}\Pi (r;s)$$

9 $${\left.\Pi \left(s\right)\right|}_{r=\infty }=0$$

where Π (*r*; *s*) is the Laplace transform of $\widehat{p}\left(r,t\right)$ and *s* denotes the Laplace transform variable.

The new variables $\Phi \left(z\right)\equiv \widehat{p}/{z}^{\gamma }$ and $z=(r+R)s/c$ transform Eqs. (8) and (9) into the modified Bessel equation

10 $${z}^{2}\frac{{d}^{2}\Phi (z)}{d{z}^{2}}+z\frac{d\Phi (z)}{dz}=({z}^{2}+{\gamma }^{2})\Phi (z)$$

with the boundary condition

11 $$\underset{z\to \infty }{lim}[{z}^{\gamma }\Phi (z)]=0$$

The general solution of Eq. (10) is

12 $$\Phi (z)=\alpha {I}_{\gamma }(z)+\beta {K}_{\gamma }(z)$$

where *α* and *β* are arbitrary constants, while ${I}_{\gamma }(z)$ and ${K}_{\gamma }(z)$ denote modified Bessel functions of order γ. Such functions have the asymptotic expansions^23^

13 $${I}_{\gamma }(z)\approx \frac{exp(z)}{\sqrt{2\pi z}}\left[1-\frac{(4{\gamma }^{2}-1)}{8z}+\frac{(4{\gamma }^{2}-1)(4{\gamma }^{2}-9)}{128{z}^{2}}-\dots \right]$$

and

14 $${K}_{\gamma }(z)\approx \sqrt{\frac{\pi }{2z}}exp(-z)\left[1+\frac{(4{\gamma }^{2}-1)}{8z}+\frac{(4{\gamma }^{2}-1)(4{\gamma }^{2}-9)}{128{z}^{2}}+\dots \right]$$

valid for large *z*. Therefore, in order that (11) be satisfied, *α* must be zero.

Hence, the solution for Π(*z*) becomes

15 $$\Pi (z)=\beta {z}^{\gamma }{K}_{\gamma }(z)$$

Differentiation of Eq. (15) yields^23^

16 $$-\frac{d\Pi (z)}{dz}=\Pi (z)\frac{{K}_{\gamma -1}(z)}{{K}_{\gamma }(z)}$$

The Bessel function ratio may be written as

17 $$\frac{{K}_{\gamma -1}(z)}{{K}_{\gamma }(z)}=1+\frac{1/2-\gamma }{z}\left[1-\frac{1/2+\gamma }{2z}(1+\dots )\right]$$

which terminates for $\gamma =\pm 1/2$ but has an infinite number of terms for γ = 0. Provided *z* > 1, the error introduced by the truncation of (17) to

18 $$\frac{{K}_{\gamma -1}(z)}{{K}_{\gamma }(z)}\approx 1+\frac{1/2-\gamma }{z}$$

is less than 5% for γ = 0^23^. Equation (18), of course, is exact for $\gamma =\pm 1/2.$

Combining Eqs. (16) and (18) and restoring the original variables leads to

19 $$-\frac{\partial \Pi }{\partial r}=\Pi \left(\frac{s}{c}+\frac{1/2-\gamma }{r+R}\right)$$

Taking the inverse Laplace transform of Eq. (19) yields

20 $$-\frac{\partial (p-{p}_{0})}{\partial r}=\frac{1}{c}\frac{\partial (p-{p}_{0})}{\partial t}+\frac{1/2-\gamma }{r+R}(p-{p}_{0})$$

Integration of Eq. (20) with respect to time gives

21 $$p\left(r,t\right)+c\frac{1/2-\gamma }{r+R}{\int }_{0}^{t}p\left(r,\zeta \right)d\zeta ={p}_{0}\left(1+\frac{1/2-\gamma }{r+R}ct\right)-c{\int }_{0}^{t}\frac{\partial p\left(r,\zeta \right)}{\partial r}d\zeta $$

Finally, recalling that $-\frac{\partial p}{\partial r}=\rho \frac{\partial u}{\partial t}$^24^,

22 $$p\left(r,t\right)+c\frac{1/2-\gamma }{r+R}{\int }_{0}^{t}p\left(r,\zeta \right)d\zeta ={p}_{0}\left(1+\frac{1/2-\gamma }{r+R}ct\right)+\rho cu\left(r,t\right)$$

where *ρ* is the density of the medium, through which sound waves propagate, and *u* is the velocity perturbation.

Equation (22) provides a relation between the local values of the acoustic pressure, *p* (*r*, *t*) and the corresponding velocity perturbation, *u*(*r*, *t*). It is valid everywhere within the domain including the boundary (surface), *r* = 0.

In case of the planar geometry, γ = 1/2, Eq. (22) reduces to a simple algebraic equation, namely:

23 $$p(r,t)={p}_{0}+\rho cu(r,t)$$

## Incorporation of the sound source function

In the most general case, the sound source function in Eq. (2) can be given in the form of Fourier series as

24 $$S\left(r,t\right)=\sum_{n=1}^{\infty }\left[{a}_{n}\left(r\right)\mathit{cos}\left({\omega }_{n}t\right)+{b}_{n}\left(r\right)sin\left({\omega }_{n}t\right)\right]$$

where ${\omega }_{n}=2\pi n/T,$ whereas *T* is a certain time horizon, such as *T* ≫ *R*/*c*.

The Fourier coefficients are

25a $${a}_{n}(r)=\frac{2}{T}{\int }_{0}^{T}S(r,\zeta )cos({\omega }_{n}\zeta )d\zeta $$

and

25b $${b}_{n}(r)=\frac{2}{T}{\int }_{0}^{T}S\left(r,\zeta \right)sin\left({\omega }_{n}\zeta \right)d\zeta $$

respectively.

Now, because Eq. (22) holds for *any r*, it is possible to assume that, instead of having the velocity perturbation *u*(*r*, *t*) together with the sound source *S*(*r*, *t*), there exist a certain effective velocity perturbation

26 $${u}_{\text{eff}}\left(t\right)=u\left(t\right)+\sum_{n=1}^{\infty }\left[{a}_{n}^{*}\mathrm{cos}\left({\omega }_{n}t\right)+{b}_{n}^{*}\mathrm{sin}\left({\omega }_{n}t\right)\right]$$

with

27a $${a}_{n}^{*}=\frac{2}{T}{\int }_{0}^{T}{S}^{*}(\zeta )cos({\omega }_{n}\zeta )d\zeta $$

and

27b $${b}_{n}^{*}=\frac{2}{T}{\int }_{0}^{T}{S}^{*}(\zeta )sin({\omega }_{n}\zeta )d\zeta $$

respectively, where ${S}^{*}(t)={\int }_{0}^{\infty }S(r,t)dr$ represents the integrated effect from the sound source.

Substituting Eq. (26) into (22), the latter becomes

28 $$\begin{array}{c}p(r,t)+c\frac{1/2-\gamma }{r+R}{\int }_{0}^{t}p\left(r,\zeta \right)d\zeta ={p}_{0}\left(1+\frac{1/2-\gamma }{r+R}ct\right)+\\ +\rho cu\left(r,t\right)+\sum_{n=1}^{\infty }\left[{A}_{n}\mathit{cos}\left({\omega }_{n}t\right)+{B}_{n}\mathit{sin}\left({\omega }_{n}t\right)\right]\end{array}$$

## Model validation

To validate the model, Eq. (22) has been numerically solved for various sets of parameters. The physical properties of the domain were set as follows: *c* = 330 m/s and *ρ* = 1.2 kg/m^3^. The reference pressure was set at *p*_0_ = 10^5^ Pa. Such a choice was made in order to be consistent with the results obtained in^24^.

Note that only the case of no sound sources was considered. This was done, in order to see the effects of the domain geometry and boundary velocity perturbations clearer. Also, due to the linearity of Eq. (2), the effect from the source can be superimposed on the solutions presented in this section.

**Figure 1:**
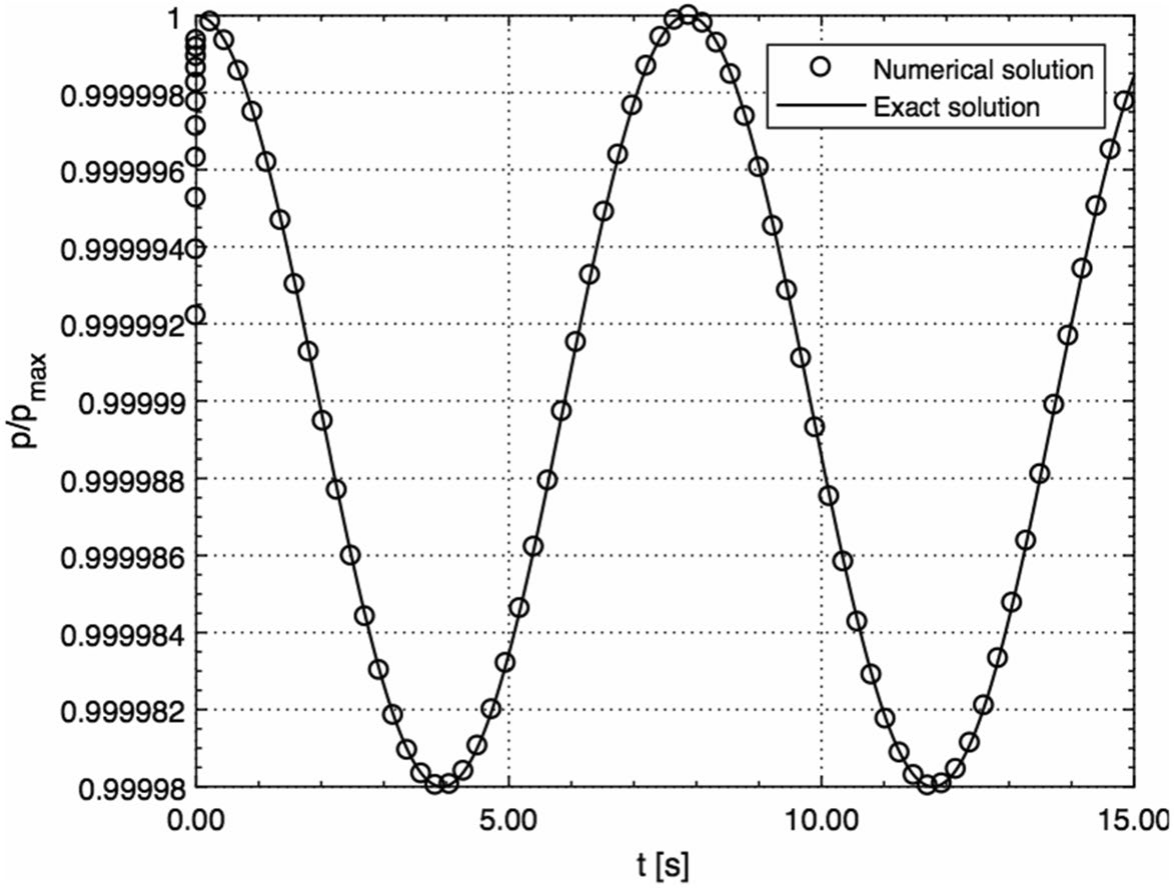
Comparison between the solution given by Eq. (22) and exact solution for the spherical sound wave with no surface (*R* = 0) obtained in^24^ (normalised by *p*_max_ = 10^5^ Pa).

Figure 1 shows a comparison between the solution given by Eq. (22) and exact solution for the spherical sound wave with no surface (*R* = 0) obtained in^24^. The velocity perturbation is given by

29 $$u(r,t)=\frac{1}{\rho c}\frac{A}{rcos\theta }cos(\omega t-kr-\theta ),k=\omega /c,\theta =arccos\left(\frac{kr}{\sqrt{1+{k}^{2}{r}^{2}}}\right)$$

for which the exact solution for the acoustic pressure follows as

30 $$p={p}_{0}+\frac{A}{r}cos(\omega t-kr)$$

where *ω* = 0.8 Hz and *A* = 1 were chosen, to match the result of^24^.

As can be seen from Fig. 1, a very good agreement between the two results is achieved.

**Figure 2:**
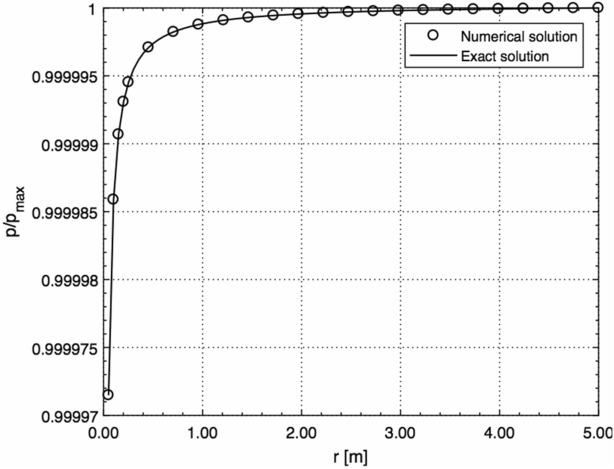
Comparison between the solution given by Eq. (22) and exact solution for the spherical sound wave with no surface (*R* = 0) obtained in^24^ in the case of different distances from the origin (*r* = 0) at a fixed time moment (normalised by *p*_max_ = 10^5^ Pa).

Figure 2 shows a comparison between the solution given by Eq. (22) and exact solution for the spherical sound wave with no surface (*R* = 0) obtained in^24^ in the case of different distances from the origin (*r* = 0) at a fixed time moment, *t* = 10 s. Again, a practically ideal agreement between the two results is observed.

**Figure 3:**
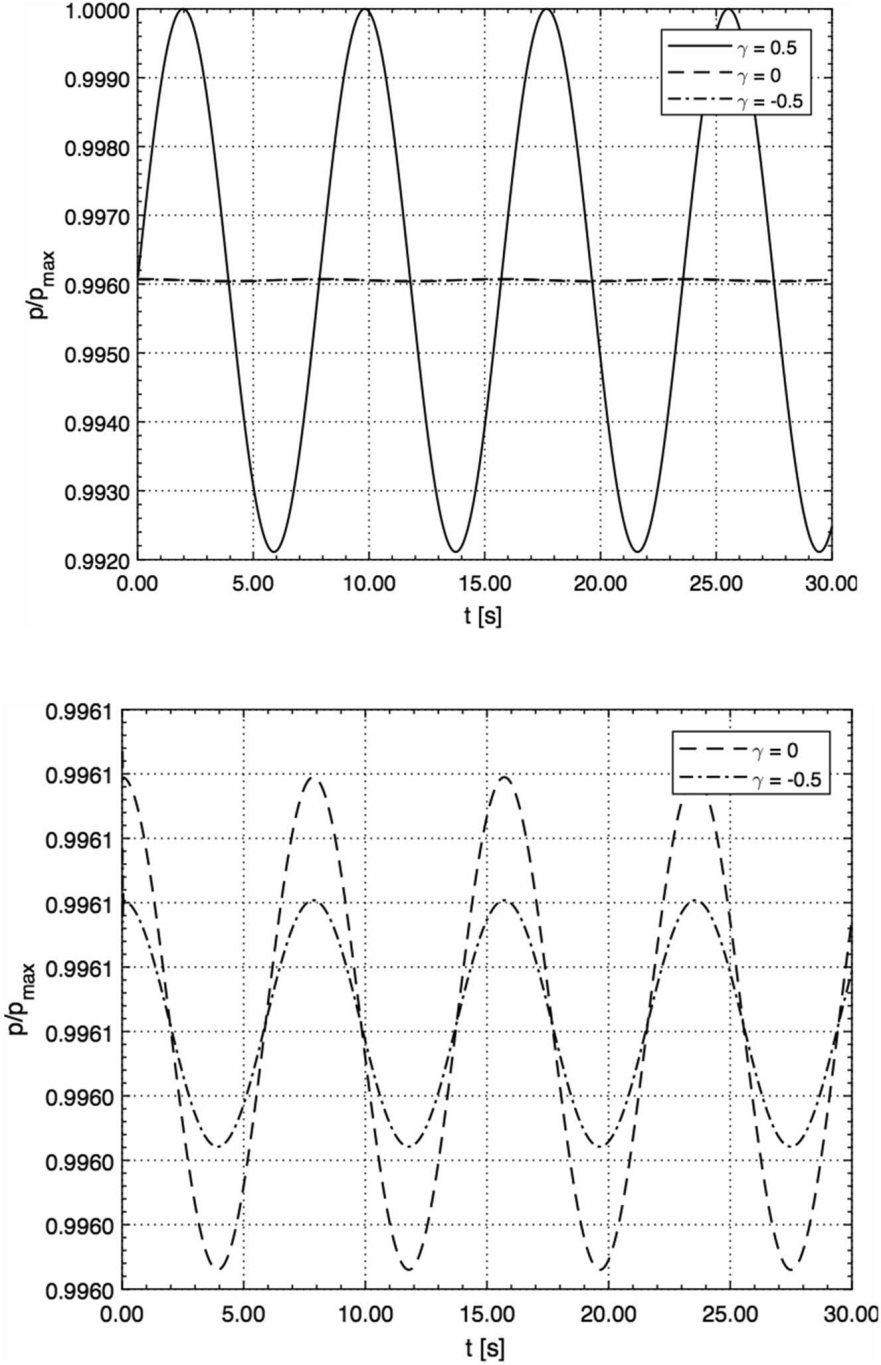
γ = 0, ± 0.5 (top); γ = 0, − 0.5 (bottom). Transient behaviour of the acoustic pressure on the surface (*r* = 0, *R* = 1 m) in the case of a periodic velocity perturbation, *u*_0_ = *u*_max_sin(*ωt*), with *u*_max_ = 1 m/s and *ω* = 0.8 Hz, for the three different geometries (normalised by *p*_max_ = 10^5^ Pa).

Figure 3 illustrates the transient behaviour of the acoustic pressure on the surface (*r* = 0, *R* = 1 m) in the case of a periodic velocity perturbation, *u*_0_ = *u*_max_sin(*ωt*), with *u*_max_ = 1 m/s and *ω* = 0.8 Hz, for the three different geometries.

As can be seen from Fig. 3, the planar geometry has a much more pronounced effect on the acoustic pressure than the curved geometries.

**Figure 4:**
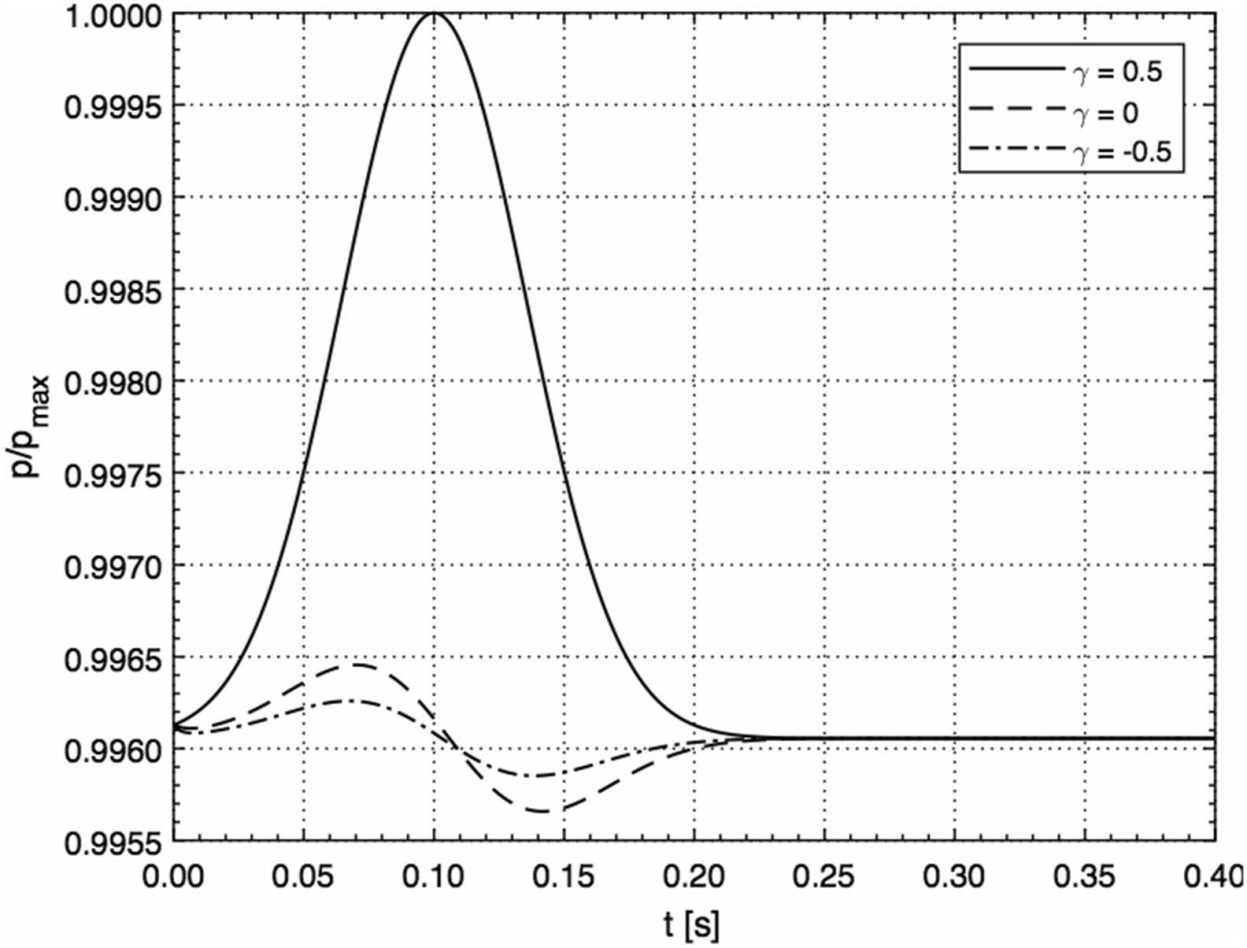
Transient behaviour of the acoustic pressure on the surface (*r* = 0, *R* = 1 m) in the case of the velocity perturbation in the form of a single pulse modelled by the Gaussian, ${u}_{0}={u}_{max}{e}^{-{\left(\frac{1-\tau }{\sigma }\right)}^{2}}$, with *u*_max_ = 1 m/s, τ = 0.1 s, and *σ* = 0.05 s, for the three different geometries (normalised by *p*_max_ = 10^5^ Pa).

Finally, Fig. 4 depicts the transient behaviour of the acoustic pressure on the surface (*r* = 0, *R* = 1 m) in the case of the velocity perturbation in the form of a single pulse modelled by the Gaussian, ${u}_{0}={u}_{\text{max}}{e}^{-{\left(\frac{t-\tau }{\sigma }\right)}^{2}}.$ with *u*_max_ = 1 m/s, τ = 0.1 s, and *σ* = 0.05 s, for the three different geometries.

As can be seen from Fig. 4, the planar geometry, in the same way as in the case of periodic velocity perturbations, has a much more pronounced effect on the acoustic pressure than the curved geometries. A possible reason for this can be that curved surfaces have larger surface areas in comparison with the planar surface of the same linear scale. Hence, lesser values of the acoustic pressure can be achieved on surfaces, which are more “curved”. Note that the lowest pressure value is always achieved in the case of spherical geometry, which is the most “curved”.

## Conclusions

It was shown that a non-field analytical method, which was previously used extensively to tackle problems in heat and mass transfer, fluid mechanics, and other areas, renders an excellent agreement with known results obtained by other, more laborious approaches. At the same time, the method provides a unified view on how the domain geometry and boundary conditions influence the transient behaviour of the acoustic pressure field.

Obviously, more studies are necessary, especially, if the method is to be employed to tackle the problems modelled by acoustic analogies^11–21^, when the sound source term plays a major role in defining the transient behaviour of the acoustic pressure field. In “Incorporation of the sound source function” of this paper, it was demonstrated that a unified approach of using the method for the acoustic analogies exists, provided the sound source functions are given in the form of the corresponding Fourier series.

The next study will be fully devoted to developing the method for tackling problems modelled by the acoustic analogies, in which the sound source functions will be represented through their corresponding Fourier series or generalised Fourier series.

## List of symbols

*A*_n_, *B*_n_: Fourier coefficients
*a*_n_, *b*_n_: Fourier coefficients
*c* (m/s): Speed of sound
*Ι*_*γ*_, *Κ*_γ_: Modified Bessel functions of order γ
*p* (Pa): Pressure
*p*_0_ (Pa): Reference pressure
$\widehat{p}$ (Pa): Acoustic pressure
*R* (m): Radius of the surface
*r* (m): Spatial variable
*S* (Pa/s^2^): Sound source function
*s* (1/s): Laplace transform variable
*T* (s): Time horizon
*t* (s): Time
*u* (m/s): Velocity perturbation
*x* (m): Spatial variable
*z*: Auxiliary variable

## Greek

*α*, *β*: Arbitrary constants
*γ*: Geometric factor
*ζ* (s): Dummy integration variable
Π (Pa): Laplace transform of the excess acoustic pressure
*ρ* (kg/m^3^): Density
*σ* (s): Variance of the velocity pulse
*τ* (s): Mean of the velocity pulse
Φ (Pa): Auxiliary variable
*ω* (1/s): Harmonic frequency

## Special symbol

∇: Laplacian
